# Reservoirs of antimicrobial resistance in the context of One Health

**DOI:** 10.1016/j.mib.2023.102291

**Published:** 2023-06

**Authors:** Milena Despotovic, Laura de Nies, Susheel Bhanu Busi, Paul Wilmes

**Affiliations:** 1 Systems Ecology Group, Luxembourg Centre for Systems Biomedicine, 7 Avenue des Hauts Fourneaux, L-4362 Esch-sur-Alzette, Luxembourg; 2 Department of Life Sciences and Medicine, Faculty of Science, Technology and Medicine, University of Luxembourg, 6, avenue du Swing, Belvaux, L-4367, Luxembourg

## Abstract

The emergence and spread of antimicrobial resistance (AMR) and resistant bacteria, are a global public health challenge. Through horizontal gene transfer, potential pathogens can acquire antimicrobial resistance genes (ARGs) that can subsequently be spread between human, animal, and environmental reservoirs. To understand the dissemination of ARGs and linked microbial taxa, it is necessary to map the resistome within different microbial reservoirs. By integrating knowledge on ARGs in the different reservoirs, the One Health approach is crucial to our understanding of the complex mechanisms and epidemiology of AMR. Here, we highlight the latest insights into the emergence and spread of AMR from the One Health perspective, providing a baseline of understanding for future scientific investigations into this constantly growing global health threat.

## Highlights

• AMR is a leading and constantly growing threat to global health.
• The human microbiome is an important AMR reservoir.
• Human–animal–environment interactions favor AMR dissemination.
• One Health is crucial for understanding AMR transmission and spread.

**Current Opinion in Microbiology** 2023, **73**:102291

This review comes from a themed issue on **Microbiota**

Edited by **Christopher Stewart** and **Maria Carmen Collado**

For complete overview of the section, please refer to the article collection, “Microbiota”

https://doi.org/10.1016/j.mib.2023.102291

1369–5274/© 2023 The Authors. Published by Elsevier Ltd. This is an open access article under the CC BY license (http://creativecommons.org/licenses/by/4.0/).

## Introduction

Globalization and enhanced mobility, a growing human population, close contact with animals and their environment, intensive farming, pollution, ecosystem degradation, and climate changes have led to the emergence of new pathogens and the spread of antimicrobial resistance (AMR). Throughout history, bacterial infections have been a major cause of human and animal diseases. The discovery and use of antibiotics enabled their effective management from a clinical point of view, but at the same time resulted in increased AMR. If no measures are undertaken to mitigate the current rates of emergence and spread of AMR, it is estimated that AMR will result in a financial burden of 100 trillion dollars at the global level and cause over 10 million deaths per year by 2050 [1].

Human health is tightly linked to the health of animals and the ecosystems they share, enabling resistant bacteria or antimicrobial resistance genes (ARGs) to spread between different human, animal, and environmental reservoirs. These phenomena have the potential to rapidly trigger a pandemic, whereby AMR is no longer constrained by either geographic or human–animal borders [2]. In view of the resulting limitations to conventional approaches for the prevention and control of infectious diseases, the One Health approach has emerged. One Health represents a transdisciplinary approach that shifts the focus from disease treatment and control to disease prevention and surveillance. By integrating research on resistant microorganisms circulating in humans, animals, and the environment, One Health is crucial to enhancing our understanding of the complex epidemiology of AMR [2].

In this review, we highlight the latest insights into the emergence and spread of AMR from the One Health perspective, thereby providing a baseline reference of understanding for future scientific investigations into this leading and constantly growing global health threat.

## Mechanisms of antimicrobial resistance

Bacteria have evolved various counteractive mechanisms to confer resistance to antimicrobial agents and assure their survival in a competitive environment [3]. Bacterial resistance can be classified as either natural or acquired. Natural resistance is either constitutively expressed in a bacterial species (i.e. intrinsic), or expressed upon exposure to antibiotics (i.e. induced) [4]. Acquired resistance refers to the acquisition of resistance-conferring genetic material through horizontal gene transfer (HGT). Employing HGT via mobile genetic elements (MGEs), bacteria can acquire ARGs through plasmid-mediated conjugation, transduction via bacteriophages, or integron-mediated transfer of genetic information between bacteria 3, 4. Alternatively, resistance can be acquired via mutations in the chromosomal DNA following antibiotic exposure [5]. Besides encoding for resistance to most, if not all, major classes of antibiotics, multiple genes conferring resistance to different antibiotic categories can be encoded on the same plasmid. This is especially evident in multidrug-resistant *Klebsiella pneumoniae* [6]. Furthermore, plasmids encoding ARGs are not only found within pathogenic bacteria, but can also be detected in commensals [7]. Additionally, the environment within bacterial biofilms, one of the common modes of microbial life, may promote HGT. In particular, it has been recently shown that pathogenic methicillin-resistant *Staphylococcus aureus* (MRSA) can transfer MGEs, which are too large to be packed into phages, to methicillin-sensitive *Staphylococcus aureus* strains by natural transformation in biofilms [8].

## Microbial reservoirs of antimicrobial resistance

**Figure 1:**
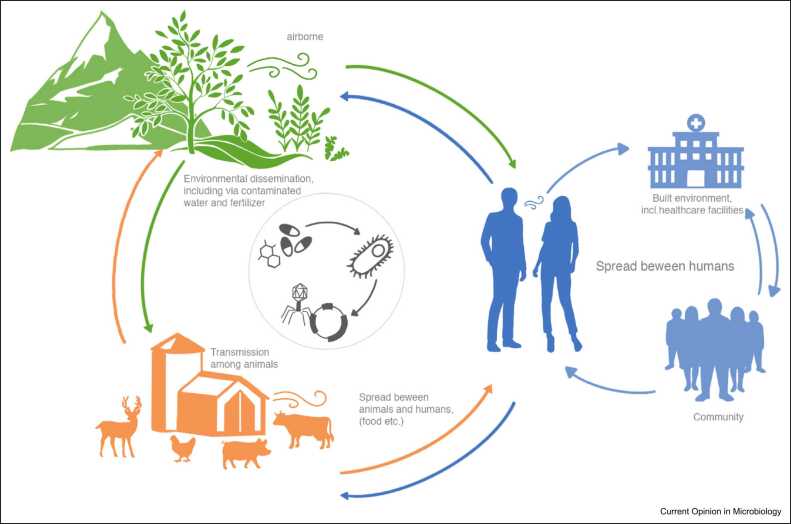
AMR dissemination in One Health. MGE-mediated (i.e. phage, plasmids, and integrons) dissemination of AMR across different microbial reservoirs.

Resistant bacteria residing within human, animal, and environmental reservoirs may spread from one to another, at both local and global levels (Fig. 1). The role of the resistome (i.e. the collection of ARGs in a given environment or organism) and the differences between ecosystems are of great importance not only to understand the AMR dissemination, but also to identify pools of potentially novel resistance mechanisms.

Many studies have focused specifically on the *Enterococcus faecium*, *Staphylococcus aureus*, *Klebsiella pneumoniae*, *Acinetobacter baumannii*, *Pseudomonas aeruginosa*, *Enterobacter* spp., and *Escherichia coli* pathogens that are highly resistant to last-resort drugs and extensively described in different microbial reservoirs [9]. Additionally, MRSA has been reported to be both human- and animal-associated with a high risk for zoonotic transmission 10, 11. Recent research has been extended to other pathogens posing a threat to human health, such as resistant *Campylobacter jejuni* for which infections have been reported in humans, animals, and the environment [12]. Similarly, multidrug-resistant *Salmonella* has been identified in human [13], animal [14], and environmental reservoirs [15].

In the context of One Health, natural microbial communities, or microbiomes, may also have an important role in the dissemination of AMR. The structure of human and animal microbiomes is shaped by several factors, including exposure to microorganisms through contacts with exogenous sources (e.g. animals, environment), specific host–microbe interactions, and the outcome of competitive, cooperative, and/or predatory (phage) interactions [16]. Recent evidence suggests that ARGs in environmental bacteria can be rapidly acquired by human-associated and pathogenic bacteria [17], thereby posing a considerable threat to human health.

### Human

The prevalence of AMR and the studies thereof have mostly been limited to clinically relevant pathogenic bacteria. These include but are not limited to extended-spectrum beta-lactamase (ESBL)-producing and carbapenem-resistant *Klebsiella pneumoniae*, ESBL-, AmpC-, and carbapenemase-producing *Escherichia coli*, carbapenem-resistant *Acinetobacter baumannii* and *Pseudomonas aeruginosa*, vancomycin-resistant *Enterococcus faecium*, MRSA, penicillin-resistant *Streptococcus pneumoniae*, as well as fluoroquinolone-resistant *Salmonella* and *Shigella* species. More recently, lesser-known human pathogens such as *Corynebacterium diphtheriae* isolates have been reported to carry penicillin, macrolide, and multidrug resistance [18]. However, AMR is also associated with the human microbiome. Although most of the microorganisms constituting the human microbiome are commensals, they have an important role in AMR dissemination. The transfer of AMR can occur from pathogenic bacteria to commensals [19], and from commensals or environmental bacteria to the members of the microbial community [20]. Once ARGs are acquired, commensal organisms may mediate the dissemination of AMR to the microorganisms with pathogenic potential. Interestingly, resistance potential in the gut microbiome exhibits significant differences between geographical areas resulting from differences in antibiotic usage as well as those linked to medicine and food production [21]. In this context, the oral cavity is an important gateway as it constantly reloads the gut microbiome by oral-to-gut transmission [22], and represents an additional microbial reservoir contributing to the resistome [23]. Similarly, due to the constant shedding of microbiota in the environment, the human skin is also an important AMR reservoir [19]. The treatment with systemic antibiotics is associated with long-lasting changes of the human skin microbiome composition potentially contributing to the AMR [24]. Additionally, bacterial transmission within the host may contribute to AMR at smaller spatial scales or in case of a low mutation rate [25]. For example, Wheatley *et al*. revealed a *Pseudomonas aeruginosa-*resistant lineage within the gut of a critically ill individual demonstrating local resistance adaptation due to the organ-specific selective pressure. Subsequently, the same strain was found in the lung suggesting within-host transmission as one of the AMR dissemination mechanisms [25].

Transmission of AMR has been extensively discussed in the context of sanitary conditions, such as open defecation or access to clean water [26]. However, recent evidence suggests that transmission of ARGs via air (i.e. bioaerosols) may lead to an increased prevalence of HGT 27, 28. The spread of bioaerosols especially came into focus with the COVID-19 pandemic that has also influenced global AMR spreading. For example, in the WHO European region, reporting on resistant *Escherichia coli* and *Streptococcus pneumoniae* was decreased during 2020, likely due to the sanitary measures implemented to prevent the spread of severe acute respiratory syndrome coronavirus 2 (SARS-CoV-2). However, *Acinetobacter* spp. and *Enterococcus faecium*, resistant bacteria usually present in the hospital environments, were more frequently observed during the same period [29].

### Animals

Antibiotics are extensively used in livestock and poultry, especially in food production, leading to microbial community compositional shifts and potential increase in ARGs. Treatments using antibiotics in the animal industry also raise the risk of emergence of resistant bacteria due to longer-term selective pressures. In this context, an increased AMR abundance was detected in the gut of farm animals (chicken, turkey, and pig) compared with wild animals (boars, foxes, and rodents) [30]. Similarly, bovine fecal and nasopharyngeal microbiome changes accompanied with increased abundance of ARGs were detected after prophylactic application of antibiotics [31]. A further five-year longitudinal study in pigs and broilers treated with antibiotics for growth promotion highlighted the concomitant increase in AMR specifically in *Enterococcus* spp. [32].

Though the emergence of resistant pathogens is a critical consideration, the spread of ARGs from animal to the human microbiome is of more immediate concern. Such spread can occur via multiple routes, including the direct transmission through food products. Multiple studies have reported food animals as a source of AMR, including multidrug-resistant *Salmonella* from poultry [33], cephalosporin-resistant *Escherichia coli* from veal calves [34], and carbapenem-resistant *Escherichia coli* from pigs 35, 36. Additionally, a number of carbapenem-resistant bacteria, including *Pseudomonas, Stenotrophomonas*, and *Myroides* species, were identified in a variety of seafood products [37], underlining the argument that nonpathogenic bacteria, regularly excluded from surveillance programs, may serve as a reservoir for AMR along food supply chains 37, 38. Furthermore, resistant bacteria may spread from animals to humans through direct contact such as in the agricultural sector [16]. For example, livestock-associated MRSA was identified in workers at an industrial livestock operation, but not in workers at an antibiotic-free livestock operation [39]. These reports underline the need for a more comprehensive analysis and monitoring of livestock reservoirs of AMR.

Extensively used concentrated animal feeding operations have also been recognized as AMR reservoirs and a source of resistant bacteria (e.g. *Enterococcus* spp., *Salmonella* spp*.*, *and Vibrio* spp*.*) in migratory birds 40, 41. They were recently discovered as a source of ESBL-producing *Escherichia coli* in Bangladesh [42]. Given the propensity for these birds to be in contact with humans in populated countries such as Bangladesh, it is likely that ARGs may in turn affect human health or likely disseminate within the human population.

### Environment

The role of human-influenced environments in sustaining and disseminating AMR is largely unexplored. Polluted environments (e.g. with heavy metals) contribute to the evolution and spread of AMR through coselection. Heavy metal contamination, for example, coelects antibiotic and metal resistance by cross-resistance, where single genetic mutation may mediate resistance to both metals and antibiotics, or co-resistance, where both metal- and antibiotic-resistance genes are localized on the same MGE [43]. The level of AMR in a specific environment is highly impacted by interactions between different environments. Built environments, for example, hospitals and extended care facilities, where bacteria are exposed to high and repeated doses of antibiotics, represent hotspots for AMR. Furthermore, sewage from both the hospital and the general population is ultimately transported to wastewater treatment plants that therefore provide a vast reservoir for AMR [44]. Importantly, the transmission of ARGs via MGEs was further highlighted recently by de Nies *et al.* who reported the segregation of ARG categories between plasmids and phages in wastewater treatment plants [45]. Recent evidence suggests that anthropogenic forcing of environments has led to increased AMR in the environment, such as Antarctica, that was previously recognized as pristine [46]. Therefore, the role and potential of the environment as a reservoir cannot be discounted in AMR stewardship in relation to the One Health triad.

## Approaches in assessing antimicrobial resistance: a One Health perspective

Traditionally, culture-based methods are used in clinical settings to investigate AMR and resistant bacteria [37] that are readily culturable using standard cultivation methods. However, sequencing-based methodologies allow for the genomic analysis of all organisms within a microbial ecosystem [47] providing a comprehensive view on all ARGs within different microbial reservoirs. Metagenomic studies that are focused on multiple microbial reservoirs still largely target only one side of the One Health triad, for example, human–animal, animal–environment, or environment–human 48, 49, 50. Nonetheless, some studies have pursued a complete One Health AMR approach 51, 52, showing a widespread occurrence of vancomycin-resistance genes in all environments, except from river sediments and drinking water [52]. Additionally, a number of ARGs corresponding to aminoglycoside, macrolide, beta-lactam, and tetracycline resistance were found to be widespread and present in almost all of the investigated environments [51].

To investigate the presence of AMR and MGEs within metagenomes, different bioinformatic workflows, read-, and *de novo* assembly-based methods [53] have been developed. In the context of One Health, it is crucial to study the prevalence and spread of AMR simultaneously. However, methods to systematically assess AMR within and between biomes have long remained elusive [54]. Tools such as MOCAT2 (metagenomics analysis toolkit) [55] and HUMAnN3 (HMP Unified Metabolic Analysis Network) [56] enable AMR gene identification, but do not provide any information with respect to MGE contextualization. To precisely address the gap in available methodologies, PathoFact [57], which genomically contextualizes ARGs, including their localization on MGEs, was developed. By combining effective study designs with computational analysis methods, PathoFact enables tracing the origins and dissemination of AMR from one reservoir to another using metagenomic sequencing coupled with *de novo* reconstruction of genomic fragments. Though available studies report the cross-reservoir similarities and likely transmission of AMR in a One Health setting, there is a need for more in-depth characterization of AMR transmission mechanisms, including methods to determine and classify transmission.

## Conclusions

AMR is an ever-present concern, not necessarily due to the use of antibiotics alone, but also due to anthropogenic impact and rapid globalization. A major challenge still faced by most One Health studies is attributing the directionality of ARGs between the different metagenomes. To accurately reconstruct the patterns of transmission, especially the directionality of transmission, approaches combining (meta)genomic data analysis, including phylogenetic analysis, with epidemiological approaches and time series, are needed. Since it is evident that AMR reservoirs may affect each other and given the potential role of human and animal microbiomes in AMR, understanding the interactions/mechanisms and role of each component contributing to the spread of AMR is a critical step in monitoring and addressing this challenge for human health and well-being. Recognizing the microbial reservoirs of AMR is an important first step toward this goal. Furthermore, combined methods incorporating the identity of ARGs, modes of transmission, and integration into the individual reservoirs, alongside crossover mechanisms, may be needed for comprehensive characterization of AMR dissemination. Given the dynamic interactions between humans, animals, and the environment, information on directionality in the One Health context will propel better understanding and management of the different reservoirs, especially in terms of anthropogenic effects. This will allow better antibiotic stewardship contributing to effective treatments using existing antibiotics.

## Ethics approval

Not applicable.

## Funding

P.W. acknowledges the 10.13039/501100000781European Research Council [ERC-CoG 863664]. L.dN. and P.W. were supported by the Luxembourg National Research Fund (FNR) [PRIDE17/11823097]. S.B.B. was supported by the Synergia grant [CRSII5_180241] through the 10.13039/501100001711Swiss National Science Foundation. P.W. and M.D. are supported by the Luxembourg Government through the CoVaLux programme.

## Consent to participate

Not applicable.

## Consent for publication

The authors consent to publication.

## CRediT authorship contribution statement

LdN designed and created the overview figure. MD, SBB, LdN, and PW conceptualized the review and contributed to the writing.

## Conflict of interest statement

The authors do not have any conflicts of interest relating to this work.

## Data Availability

No data were used for the research described in the article.
